# Nose size indicates maximum penile length

**DOI:** 10.1186/s12610-021-00121-z

**Published:** 2021-02-04

**Authors:** Hiroshi Ikegaya, Motofumi Suzuki, Hiroki Kondou, Taketo Kawai, Yusuke Sato, Tadaichi Kitamura, Haruki Kume

**Affiliations:** 1 Department of Forensics Medicine, Graduate School of Medicine, Kyoto Prefectural University of Medicine, Kajii-cho 465, Kawaramachi-Hirokoji, Kamigyo-ku, Kyoto, 602-8566 Japan; 2 Department of Urology, Tokyo Metropolitan Bokutoh Hospital, 4-23-15 Kotobashi, Sumida-ku, Tokyo, 130-8575 Japan; 3 Department of Urology, Graduate School of Medicine, The University of Tokyo, 7-3-1 Hongo, Bunkyo-ku, Tokyo, 113-8655 Japan

**Keywords:** Stretched penile length, Nose size, Aging, Cadaver, Longueur du Pénis étiré, Taille du Nez, Vieillissement, Cadavre

## Abstract

**Background:**

In a previous report, we investigated whether the size of male genitalia similarly exposed to serum testosterone during aging could change with age and found that penile length almost stopped increasing during adolescence and decreased in older males. In this report, to determine what factors other than age are related to penile length, we performed a multivariate analysis of the relationships between stretched penile length (SPL) and other measurements of genital organs, nose size, height and body weight in 126 adults in their 30s–50s.

**Results:**

The most highly correlated factor with SPL was flaccid penile length (*r* = 0.565, *P* < 0.0001). The next highest correlation was nose size (*r* = 0.564, *P* < 0.0001). The penile stretched rate correlated with FPL (*r* = − 0.690, *P* < 0.0001) but not with SPL or penile circumference.

**Conclusions:**

The fact that nose size is related to SPL indicates that penile length may not be determined by age, height or body weight but has already been determined before birth.

## Résumé

**Contexte:**

Dans un précédent article, nous avons examiné si la taille des organes génitaux masculins exposés de façon similaire à la testostérone sérique pendant l’avancement en âge pourrait changer avec l’âge; nous avons constaté que la longueur du pénis avait presque cessé d’augmenter à la période de l’adolescence et avait diminué chez les hommes plus âgés. Pour déterminer quels facteurs autres que l’âge étaient liés à la longueur du pénis, nous avons réalisé, dans la présente étude, une analyse multivariée des relations entre la longueur du pénis étiré (LPE) et d’autres mesures des organes génitaux, de la taille du nez, de la taille et du poids corporels chez 126 adultes âgés de 30 à 50 ans.

**Résultats:**

Le facteur le plus fortement corrélé avec la longueur du pénis étiré était la longueur du pénis flasque (*r* = 0.565, *p* < 0.0001). La corrélation suivante la plus élevée était la taille du nez (*r* = 0,564, p < 0,0001). Le taux d’étirement du pénis était corrélé à la longueur du pénis étiré (*r* = − 0.690, *p* < 0.0001) mais n’était corrélé ni à la longueur du pénis flasque ni à la circonférence du pénis.

**Conclusions:**

Le fait que la taille du nez soit liée à longueur du pénis étiré indique que la longueur du pénis peut ne pas être déterminée par l’âge, la taille ou le poids corporels, mais qu’elle a déjà été déterminée avant la naissance.

## Background

Many unidentified dead bodies are discovered every day, not just from acts of terrorism and natural disasters, and this is becoming a major problem worldwide [1]. In Japan, with a population of 120 million, approximately 1000 people are discovered annually as unidentified cadavers, and the police are required to investigate these cases.

In their initial investigations of unidentified corpses, the police seek to identify them using factors, such as age and gender, as well as by their belongings. These dead bodies are often thought to be skeletons or highly decayed. However, in fact, most of them are recently deceased.

Gender is relatively easy to determine by assessing the genitals or DNA examination. However, it is very difficult to estimate age based on cadaver appearance.

Recently, some researchers reported the utility of computed tomography (CT) images for age estimation, especially for the living or nonskeletonized corpses [2, 3]. However, a body must be transported to obtain CT images, which requires time and effort and incurs costs.

We previously reported that postmortem serum prostate-specific antigen (PSA) level and prostate volume correlate with age and discussed the use of PSA in an objective age estimation [4].

The age-related increase in serum PSA levels has been attributed mainly to prostate enlargement due to prolonged exposure to serum testosterone during aging [5–7]. Testosterone is also related to the growth of the penis in newborn infants [8].

Therefore, we also investigated whether the size of the male genitalia similarly exposed to serum testosterone during aging could change with age in our previous report. We found that penile length almost stopped increasing during adolescence, remained unchanged in middle age, and decreased slightly in older males [9]. The saying “Big nose, big hose” suggests that nose size indicates penile length. However, scientific reports that prove this statement are not available to date.

In this report, to confirm the truth of this statement, we analyzed the relationships between stretched penile length (SPL) and nose size as well as the weight of male genital organs in middle-aged people.

## Materials and methods

**Fig. 1:**
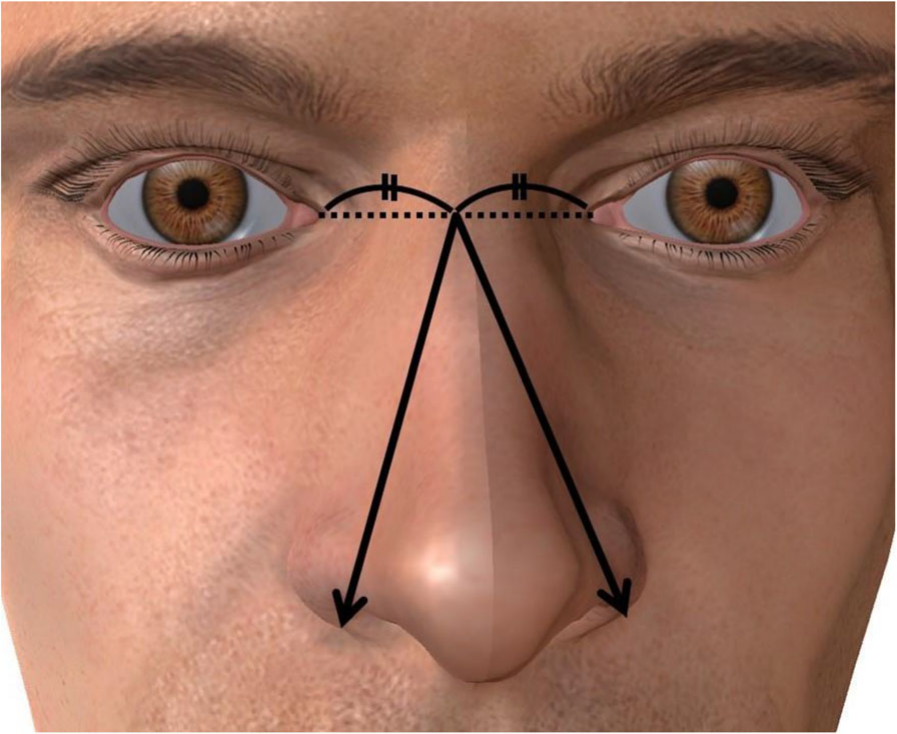
The measurement of nose size. Nose size was defined as the longer distance between the midpoint of the left and right medial ocular angles and the outside of the left or right nose wings (indicated by arrow)

One hundred twenty-six male forensic autopsy cases at Kyoto Prefectural University of Medicine from April 2015 to March 2019 were used in this study. All cases were in their 30s–50s, and their postmortem time was within 3 days. Height, weight, SPL, flaccid penile length (FPL), penile circumference (PC), right and left testicular weights, prostate weight, and nose size were measured at the autopsy by one researcher during the period. SPL was measured by manual traction [10]. SPL was measured at room temperature, which was maintained at 22 °C, with the cadaver lying in the supine position and the penis placed at a 90° angle to the body. The nose size was defined as the longer distance between the midpoint of the left and right medial ocular angles and the outside of the left or right nose wings (Fig. 1).

This study was approved by the Institutional Review Board of Kyoto Prefectural University of Medicine (ERB-C-1491-1).

### Statistical analysis

Relationships among age, height, body weight and factors related to the genital organs were analyzed using Pearson’s linear correlation. To identify independent predictive factors influencing SPL, multivariate analyses were performed using linear regression models. Analysis was performed using JMP software (SAS Institute Inc., Cary, NC).

## Results

Table 1 presents the relationships among age, height, body weight and factors related to the genital organs.

**Table 1:**
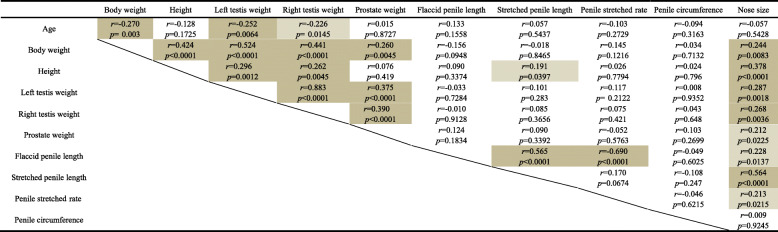
Relationships among age, height, body weight and factors related to genital organs (multivariate analysis using linear regression models)

*r* Pearson correlation coefficient. *p* Dark gray-hatched cells represent *p* < 0.010). Gray cells indicate *p* < 0.050)

The most highly correlated factors were stretched penile length and flaccid penile length (*r* = 0.565, *p* < 0.0001). The next highest correlation was noted for stretched penile length and nose size (*r* = 0.564, *p* < 0.0001). The penile stretch rate was highly correlated with the flaccid penile length (*r* = − 0.690, *p* < 0.0001) but not with the stretched penile length or penile circumference. Nose size was correlated with all factors except penile circumference: height (*r* = 0.378, *P* = 0.083), body weight (*r* = 0.378, *P* < 0.0001), left testis (*r* = 0.287, *p* < 0.0018), right testis (*r* = 0.268, *p* = 0.004), prostate weight (*r* = 0.212, *p* = 0.0225), flaccid penile length (*r* = 0.228, *p* = 0.137) and nose size. Body weight was highly correlated with height (*r* = 0.424, *p* < 0.0001), left testis (*r* = 0.524, *p* < 0.0001), right testis (*r* = 0.441, *p* < 0.0001), prostate weight (*r* = 0.260, *p* = 0.0045) and nose size but not with stretched penile length, flaccid penile length or penile circumference. Age was weakly correlated with body weight (*r* = − 0.270, *p* = 0.003), left testis (*r* = − 0.252, *p* = 0.0064) and right testis (*r* = − 0.226, *p* = 0.0145).

**Fig. 2:**
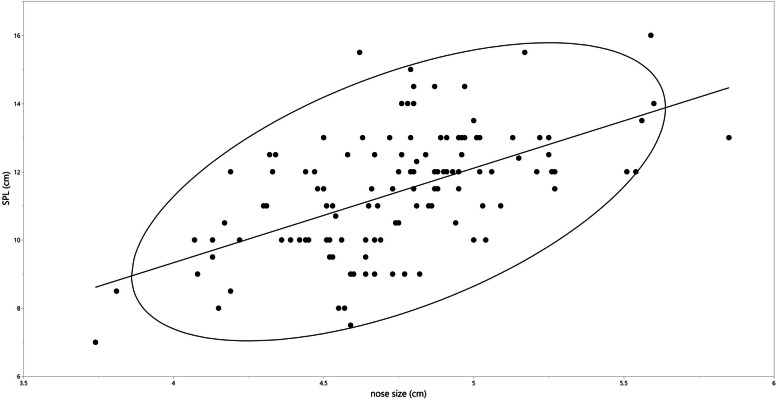
Linear regression analysis of stretched penile length and nose size. SPL: stretched penile length

The linear regression model is presented in Fig. 2. The relationship between stretched penile length and nose size is presented in Table 2. The average stretched penile length gradually increased in the longer nose size groups.

**Table 2:** Relationship between nose size and stretched penile length

Nose size (cm)	n	Average SPL (cm)	SD
−4.50	27	10.37	1.56
4.51–5.00	75	11.40	1.77
5.01–5.50	18	12.33	1.16
5.51−	6	13.42	1.50

*SPL* Stretched penile length, *SD* Standard deviation, *n* Number

## Discussion

In our study, nose size was not correlated with factors related to body physiques but was correlated with SPL. In previous reports, embryonic androgens and androgen receptors were associated with penile growth [11, 12]. Penile growth is also consistent with SPL being associated with the second to fourth digit ratio, which has been linked to androgen exposure [13]. In the field of forensic medicine, it is widely known that the size of the skull, excluding the jawbone, is relatively unaffected by aging. Similar to the SPL, as a representative measurement of head parts, nose size may be also not determined by age or body size but has already been determined before birth.

Our study also suggests that SPL and nose size can be used as indicators independent of age and postnatal body size.

As expected, a significant correlation was found between SPL and FPL. This finding is attributed to the fact that a person with a longer FPL may naturally have a longer SPL given the flexibility of penile tissue. Contrary to our data, showing that FPL was negatively correlated with penile stretched rate would suggest that the individual had a different penile extension rate. The same result was also noted in another report [13]. The elasticity of a small, flaccid penis may be greater than that of a large, flaccid penis.

In contrast to SPL, a weak correlation with nose size was found with FPL (*r* = 0.228, *p* = 0.0137). This finding may be attributed to the fact that the contraction rate of the penis varies slightly based on FPL in contrast to SPL, which indicates the maximum length during erection. In addition, the absence of blood flow in the cadavers contributed to the variation in the measured values of FPL.

A high second to fourth digit ratio may indicate a small penis [13]. In addition, there is a report that a high second to fourth digit ratio may indicate small testes [14]. In addition, a correlation may exist between testis weight and nose size. Unfortunately, we did not analyze the second to fourth digits in our present study. Only weak correlations were noted between left and right testis weights and nose size in our study.

Although our results are useless for forensic purposes, understanding the growing process of the penis or facial features may be very important for extrapolating fetal androgen levels and following male genital functions. This study is the first to demonstrate the relationship between SPL and nose size but is limited in Japanese male cadavers, and the reason why SPL and nose size are related is still unclear. Therefore, we consider it an interesting subject to pursue from now on.

## Conclusions

Nose size was highly related to stretched penile length in Japanese male cadavers.

## Data Availability

The datasets generated during and/or analyzed during the current study are available from the corresponding author on reasonable request.

## Abbreviations

BMI: Body mass index
CT: Computed tomography
FPL: Flaccid penile length
PC: Penile circumference
PSA: Prostate-specific antigen
SPL: Stretched penile length
